# Remote Ischemic Conditioning May Improve Disability and Cognition After Acute Ischemic Stroke: A Pilot Randomized Clinical Trial

**DOI:** 10.3389/fneur.2021.663400

**Published:** 2021-08-30

**Authors:** Alina Poalelungi, Delia Tulbă, Elena Turiac, Diana Stoian, Bogdan Ovidiu Popescu

**Affiliations:** ^1^Department of Neurology, Emergency Clinical Hospital, Bucharest, Romania; ^2^Department of Clinical Neurosciences, “Carol Davila” University of Medicine and Pharmacy, Bucharest, Romania; ^3^Department of Neurology, Colentina Clinical Hospital, Bucharest, Romania; ^4^Colentina–Research and Development Center, Colentina Clinical Hospital, Bucharest, Romania; ^5^Department of Radiology, Emergency Clinical Hospital, Bucharest, Romania; ^6^Laboratory of Cell Biology, Neurosciences and Experimental Myology, “Victor Babeş” National Institute of Pathology, Bucharest, Romania

**Keywords:** remote ischemic conditioning, acute ischemic stroke, neuroprotection, cognition, infarct size, disability

## Abstract

**Background and Aim:** Remote ischemic conditioning is a procedure purported to reduce the ischemic injury of an organ. This study aimed to explore the efficiency and safety of remote ischemic conditioning in patients with acute ischemic stroke. We hypothesized that remote ischemic conditioning administered from the first day of hospital admission would improve the infarct volume and clinical outcome at 180 days.

**Material and Methods:** We performed a unicentric double-blind randomized controlled trial. We included all patients consecutively admitted to an Emergency Neurology Department with acute ischemic stroke, ineligible for reperfusion treatment, up to 24 hours from onset. All subjects were assigned to receive secondary stroke prevention treatment along with remote ischemic conditioning on the non-paretic upper limb during the first 5 days of hospitalization, twice daily - a blood pressure cuff placed around the arm was inflated to 20 mmHg above the systolic blood pressure (up to 180 mmHg) in the experimental group and 30 mmHg in the sham group. The primary outcome was the difference in infarct volume (measured on brain CT scan) at 180 days compared to baseline, whereas the secondary outcomes included differences in clinical scores (NIHSS, mRS, IADL, ADL) and cognitive/mood changes (MoCA, PHQ-9) at 180 days compared to baseline.

**Results:** We enrolled 40 patients; the mean age was 65 years and 60% were men. Subjects in the interventional group had slightly better recovery in terms of disability, as demonstrated by the differences in disability scores between admission and 6 months (e.g., the median difference score for Barthel was −10 in the sham group and −17.5 in the interventional group, for ADL −2 in the sham group and −2.5 in the interventional group), as well as cognitive performance (the median difference score for MoCA was −2 in the sham group and −3 in the interventional group), but none of these differences reached statistical significance. The severity of symptoms (median difference score for NIHSS = 5 for both groups) and depression rate (median difference score for PHQ-9 = 0 for both groups) were similar in the two groups. The median difference between baseline infarct volume and final infarct volume at 6 months was slightly larger in the sham group compared to the interventional group (*p* = 0.4), probably due to an initial larger infarct volume in the former.

**Conclusion:** Our results suggest that remote ischemic conditioning might improve disability and cognition. The difference between baseline infarct volume and final infarct volume at 180 days was slightly larger in the sham group.

## Introduction

Stroke is one of the leading causes of mortality and morbidity worldwide, with significant global burden and costs (1). It is the first cause of disability and the second cause of cognitive decline (2). Nevertheless, the only approved treatment for acute ischemic stroke (AIS) (which accounts for 87% of all strokes) (1) is reperfusion therapy (intravenous thrombolysis with alteplase and/or mechanical thrombectomy) (3). Unfortunately, in Romania the treatment of AIS remains extremely limited, mainly because patients do not recognize stroke symptoms/signs and arrive late to the hospital, therefore missing the reperfusion therapeutic window. In these cases, there is a great need for neuroprotective interventions in order to improve the outcome of AIS.

Over the last 20 years, many neuroprotective agents and interventions have been studied. Remote ischemic conditioning (RIC) is a new area of interest in stroke and neuroprotection (4). It is a potential non-invasive intervention meant to induce transient and brief periods of ischemia remote from the ischemic injury site. In AIS, single or repeated cycles of transient limb(s) ischemia followed by reperfusion are employed, usually with a blood pressure cuff inflated to a level above the systolic blood pressure for a few minutes, followed by deflation. This stems from the hypothesis that RIC could prevent cerebral damage after AIS by preventing/reducing the ischemia-reperfusion injury (neuroprotection).

Ischemia-reperfusion injury resulting from arterial occlusion in ischemic stroke is characterized by metabolic dysfunction, apoptosis, necrosis, and local inflammatory processes (5). The neuroprotective mechanism of RIC is not clearly understood, but it has been suggested that it is mediated by humoral, neuronal, and inflammatory pathways (6). Remote ischemic conditioning seemingly involves the transfer of a humoral substance from one organ to another. It triggers the release of humoral factors and local autocoids (adenosine, bradykinin, and gene-related peptide) which activate neurogenic transmission (with involvement of muscle afferents and autonomic nervous system) and involve immune pathways by suppressing proinflammatory genes in immune cells. Furthermore, RIC reduces oxidative damage and suppresses the inflammatory responses in the brain. This mechanism can last days after revascularization. More details can be found in excellent detailed reviews about this topic (4, 7, 8).

Ischemic conditioning was introduced in Cardiology in 1986 by Murry et al. who performed short repetitive occlusion/reperfusion of the coronary artery in canine models and observed a significant reduction in infarct size (9). Subsequent studies showed that ischemic conditioning can also be applied remote from the ischemic injury site. Brief episodes of ischemia-reperfusion in a distant organ (e.g., upper or lower limb) were proposed to exert a neuroprotective effect (10). Various studies indicated that limb RIC is neuroprotective in animal models of stroke (11, 12).

In AIS, RIC can be applied prior to the ischemic event (remote ischemic preconditioning), during the ischemic event (remote ischemic perconditioning), or after the vascular event (remote ischemic postconditioning). A mechanical tourniquet or automatic device placed around an arm or leg is meant to perform repetitive cycles of inflation and deflation. Most of the clinical studies chose to perform 3 to 5 cycles of transient limb ischemia, with different duration of mechanical vessel occlusion, ranging from 3 to 5 min. The most frequent site of limb conditioning was a single-arm (13). These protocols were influenced by the Cardiological ischemic conditioning protocols, but currently it is unknown whether these differences modify the efficacy of RIC. The major advantage of remote ischemic perconditioning is the broad therapeutic window- although time-sensitive, it can be carried out even after exceeding the therapeutic window for reperfusion treatment (thrombolysis/thrombectomy).

There is a limited number of clinical trials evaluating the efficacy of RIC in the treatment AIS. The majority of these ongoing trials explore the benefits, feasibility, and risks of applying RIC as soon as possible (even from the ambulance) after AIS onset (14). In particular, they focus on the effect of RIC on clinical outcome scales and final infarct volume.

From all the clinical trials employing RIC in patients with AIS, Purroy F et al. identified only four randomized controlled trials with completed and published data (15). The first study on RIC in AIS was conducted by Hougaard KD et al. in 2014 (14). They tested the effect of RIC vs. sham performed during the prehospital phase (in the ambulance) in AIS patients, in conjunction with thrombolysis. The primary outcome was the penumbra salvage, with a follow-up at 90 days. Overall, a final infarct volume analysis suggested that prehospital RIC might have immediate neuroprotective effects (14). In the RECAST and RECAST-II, England et al. investigated the effect of RIC vs. sham in patients with hyperacute AIS (26 patients recruited within 24 h of stroke onset and 60 patients enrolled within 6 h of stroke onset, respectively) by inducing transient non-paretic arm ischemia through a manual standard blood pressure cuff (16, 17). The primary outcome was feasibility, whereas the second outcome included functional outcomes. The duration of follow-up was 90 days. The conclusion was that RIC applied twice daily is feasible, well-tolerated, with no serious adverse events, and good adherence in hyperacute stroke (16, 17). Che et al. investigated and demonstrated the feasibility and safety of arm RIC after thrombolysis in 30 patients with AIS (18). The multicenter RESCUE BRAIN trial conducted by Pico et al. inquired whether leg RIC reduces final ischemic volume after AIS. They included 188 patients with confirmed carotid ischemic stroke within 6 hours of symptoms onset and concluded that treatment with RIC during and after AIS reperfusion therapy does not significantly reduce the brain infarct volume growth (19). Considering recent data and evidence, RIC is a simple, inexpensive, well-tolerated intervention, posing minimal risk. Clinical trials have demonstrated the feasibility of delivering RIC at different stages of hospitalization for AIS.

In 2018, Zhao et al. conducted a meta-analysis aiming to assess the benefits and harms of RIC in preventing or treating AIS (20). They included seven randomized controlled studies of RIC vs. sham in subjects with either AIS, chronic cerebral ischemia (i.e., 14 days after symptoms onset)/gradual onset cerebral ischemia, or intracranial/extracranial moderate/severe stenosis/confirmed occlusion, encompassing different protocols for RIC. Out of these, three trials on the effects of RIC on ischemic stroke prevention were included in the analysis, whereas four trials analyzed the effects of RIC on ischemic stroke treatment. The latter category included two studies enrolling patients with cerebral small vessel disease and two studies with AIS subjects. By combining their results, the overall effect (i.e., stroke severity- final infarct volume and clinical scores) was not significantly different between the intervention and sham group in AIS patients (20).

Since clinical trials have employed different RIC protocols in AIS patients, their cumulative results should be interpreted with caution. Temporal inclusion criteria had great variability. Remote ischemic conditioning was initiated during transportation to the hospital or immediately after admission. It was employed isolated or as add-on therapy to revascularization (alteplase or endovascular methods). The procedure was also different among studies, with various repetitions per day or number of days of RIC, possibly responsible for the inconsistent results. Notably, there were significant differences in main endpoints. Some studies focused on final infarct volume, whereas others addressed clinical stroke severity after RIC. Further trials with uniform and standardized protocols might fill in these gaps and provide better knowledge of RIC mechanisms and effects.

Our study aims to explore the efficiency and safety of RIC applied to the non-paretic upper limb in patients with AIS ineligible for reperfusion treatment. We hypothesize that RIC administered from the first day of AIS improves the final infarct volume and clinical outcome at 6 months. Details of the trial design have already been published (21).

## Materials and Methods

### Trial Design

We conducted a unicentric double-blind randomized controlled trial that took place between July 2018 and June 2020. It included all patients consecutively admitted to the Neurology Department of the Emergency Clinical Hospital from Bucharest, Romania, who fulfilled the inclusion criteria. In the original trial design (21), the study was intended to be bicentric, also involving the Neurology Department of Colentina Clinical Hospital from Bucharest, Romania. Nevertheless, since Colentina Clinical Hospital became a COVID-19 support hospital (i.e., a second line hospital that admits patients with comorbidities and SARS-CoV-2 infection in order to support the first line hospitals—infectious disease and pulmonology hospitals) from May 16, 2020, it was excluded from enrollment. The study complied with the Declaration of Helsinki and was approved by the local research ethics committee.

### Subjects

We enrolled forty patients aged 50–80 years with AIS who were not eligible for thrombolysis and/or mechanical thrombectomy (e.g., patients who did not fulfill the inclusion criteria required for treatment with intravenous alteplase such as the onset of symptoms <4.5 h before beginning treatment or patients with one or more exclusion criteria such as ischemic stroke in the previous 3 months, etc.), with National Institutes of Health Stroke Scale (NIHSS) ranging from 5 to 25 points. Exclusion criteria included premorbid dependency [modified Rankin Scale (mRS) <3], significant comorbidity with any serious diseases, and life expectancy of fewer than 6 months (Table 1). Uncontrolled blood pressure (>200/100 mmHg) was also counted as an exclusion criterion. Informed consent was obtained from each patient or legal representative if the patient was unable to consent.

**Table 1:** The inclusion and exclusion criteria.

**Inclusion criteria**	**Exclusion criteria**
Age: 50–80 years	Reperfusion treatment (intravenous thrombolysis, mechanical thrombectomy)
5 ≤ NIHSS ≤ 25	5 > NIHSS > 25
Ischemic stroke confirmed by CT scan	Hemorrhagic stroke on CT scan
<24 h from onset to treatment	Fluctuating neurological deficit
Signed informed consent	Transient ischemic attack
	Other cerebral lesions: cerebral tumors, arteriovenous malformation
	Uncontrolled blood pressure (<90/60 or > 200/100 mmHg)
	Difference between systolic blood pressure in the upper arms > 10 mmHg
	Ischemic events in the last 6 months (AIS, myocardial infarction, etc.)
	Premorbid mRS > 3
	Comorbidity with any serious disease and life expectancy of <6 months.

All patients meeting the clinical criteria for stroke underwent brain CT scan at baseline and follow-up at 6-months. The clinical examination and neurological scales were performed at baseline, 3 and 6 months.

All patients continued the standard treatment for secondary prevention of AIS according to the national guidelines.

### Randomization and Intervention

The subjects were allocated in a double-masked and randomized fashion 1:1 to either the experimental or control group. The randomization was performed by predefined drawing lots from a large number of sealed opaque envelopes containing the RIC instructions.

A manual tourniquet was placed around the upper limb contralateral to the neurological deficit in the first 24 hours from the onset of symptoms/signs suggestive of AIS. All patients received five consecutive cycles of blood pressure cuff inflation lasting 3 min, each followed by 5 min of reperfusion, twice daily, during the first 5 five days of hospitalization (in the morning and afternoon). In the RIC group, the target of cuff inflation was 20 mmHg above the systolic blood pressure, up to 180 mmHg. Although we included patients with systolic blood pressure up to 200 mmHg, we chose the threshold of 180 mmHg (which would not induce complete restriction of blood flow in these patients) due to safety reasons, to avoid acute limb ischemia. The sham group received cuff inflation up to 30 mmHg, thus inducing sham conditioning. The procedure was performed by one of the authors (A.P.). Patients could stop the RIC treatment at any time. The patients and the medical staff who processed the data were blinded to the intervention allocation.

All patients underwent head CT scan on days 3–4 and at 180 days in the same hospital, using the same CT scanner (128 slices GE Optimal CT and 128 slices Siemens Somaton CT), unenhanced helical acquisitions, cerebral window, 140 KW(Siemens)/120 KW(GE), collimation 128 × 0.6 mm, 1.5 mm slice thickness (Siemens)/1.25 mm slice thickness (GE). The final ischemic volume was determined by manual contouring on axial CT images slice by slice, using dedicated software (GE version ADV 4.6). The CT scan at 6 months was performed in the same conditions as the first one. The reader of the CT scans was blinded to treatment allocation.

### Primary Outcome

Changes in brain lesion were evaluated by infarct volume on head CT at baseline (days 3–4) and 180 days in both groups. We acknowledge that the baseline CT is not a pre-RIC CT, but we wanted to identify a measurable infarct volume in order to calculate the difference at 6 months. We also performed a brain CT at admission in order to exclude hemorrhagic stroke, but we did not include it as a variable in the statistical analysis. We chose CT scan because it is more accessible, faster, and less expensive than MRI scanning.

### Secondary Outcome

Changes in clinical stroke severity and disability were assessed by NIHSS, mRS, Barthel Index, Lawton Instrumental Activities of Daily Living (IADL), and Katz Activities of Daily Living (ADL), whereas cognitive performance was evaluated by Montreal Cognitive Assessment (MoCA) and depression occurrence by Patient Health Questionnaire-9 (PHQ-9). All the scales were recorded at baseline, 90 and 180 days in both groups in the same Neurology Department, under similar conditions. We also recorded demographics and vascular risk factors (as defined by the World Health Organization), possible complications related to tolerance and side effects of RIC (local pain or bruising at the cuff side), cerebral complications (cerebral edema, recurrence of stroke, or other vascular events), and systemic complications (bedsores, urinary tract infection, bronchopneumonia, or death rate).

### Statistical Analysis

Statistical data were analyzed with SPSS version 20.0 Software. Categorical variables were reported as frequency and analyzed with Chi-square test. Continuous variables were reported as median (minimum, maximum) and analyzed with non-parametric tests (Mann-Whitney *U* test) provided that neither of them had normal distribution (priorly tested by Kolmogorov-Smirnov test). Hypothesis testing was 2-tailed and statistical significance was defined as *p* <0.05. Unavailable or unobtainable information (such as MoCA and PHQ-9 in aphasic patients) was recorded as missing values and excluded from the statistical analysis. According to the sample size estimation (for a mean infarct volume difference = 1.814 in the RIC group, 13.416 in the sham group, Alpha = 0.05, Beta = 0.2, Power = 0.8), 60 patients were needed: 30 in the RIC group and 30 in the sham group.

## Results

**Figure 1:**
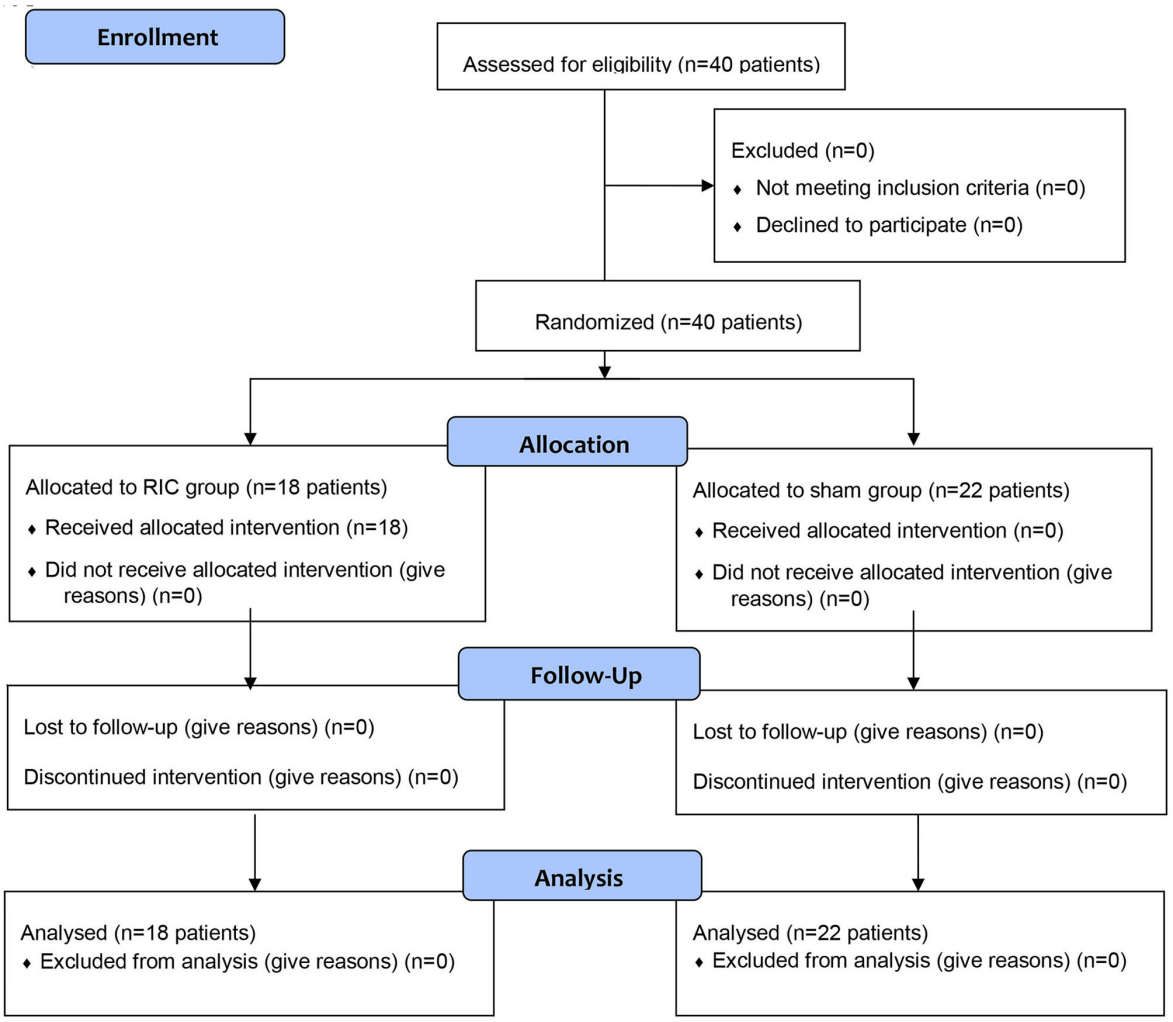
CONSORT flow chart showing the selection process of patients.

Forty patients were included in our study throughout 24 months. Eighteen patients were randomized into the RIC group and twenty-two were included in the sham group (Figure 1). The mean age was 65 years and 60% were men. Because of logistic problems related to the current SARS-CoV2 pandemic (please see above), one of the hospitals designed for enrollment was not available, therefore we could not include all the patients required by sample size estimation.

At hospital admission, the mean values of vital signs were as follows: systolic blood pressure = 154 mmHg, diastolic blood pressure = 85 mmHg, heart rate = 80 bpm, oxygen saturation = 97%, and blood glucose level = 136 mg/dl.

Sixty percent of patients were taking antihypertensive drugs, 35% antiplatelet drugs, 10% oral anticoagulants (7.5% vitamin K antagonists and 2.5% novel oral anticoagulants), and 22.5% statins.

At admission, the mean values of scales for the severity of symptoms, disability, and cognitive function were as follows: NIHSS = 7.7, mRS = 2.9, Barthel = 48, ADL = 4.3, IADL = 3.8, MoCA = 15.5, PHQ-9 = 6.2. The average infarct volume on CT was 17.5 cm^3^. The most frequent localization of AIS was in the middle cerebral artery territory (75%), followed by basilar artery (12.5%), vertebral artery (5%), and multi-territorial (7.5%). According to the TOAST classification, the most frequent etiology of AIS was large artery atherosclerosis (60%), cardioembolism (13%), small vessel occlusion (15%), and undetermined etiology (13%).

Baseline characteristics were similar in the interventional and sham group (Table 2). Concerning medical history/comorbidities, diabetes mellitus was more prevalent in the sham group (36.4 vs. 11.1%, *p* = 0.067) compared to the RIC group. Sedentarism was also more frequent in the sham group (77.3 vs. 44.4%, *p* = 0.033), reaching statistical significance.

**Table 2:** Baseline characteristics in the two groups.

**Characteristics (total *n* = 40)**	**RIC *n* = 18**	**Sham *n* = 22**	***p*-value**
Age, years	66.78 ± 6.44	64.41 ± 9.02	0.279
Male sex, *N* (%)	11 (61.11%)	13 (59.09%)	0.897
BMI, kg/m^2^	27.62 ± 4.13	29.9 ± 4.09	0.471
Abdominal circumference, cm	100.5 ± 12.72	108.5 ± 11.28	0.751
**Medical history**			
Arterial hypertension, *N* (%)	12 (66.7%)	19 (86.4%)	0.138
Diabetes mellitus, *N* (%)	2 (11.1%)	8 (36.4%)	0.067
Atrial fibrillation, *N* (%)	2 (11.1%)	3 (13.6%)	0.810
Prior stroke, *N* (%)	2 (11.1%)	2 (9.1%)	0.832
Alcohol consumption, *N* (%)	7 (38.9%)	6 (27.3%)	0.435
Smoking, *N* (%)	9 (50%)	10 (45.5%)	0.775
Sedentarism, *N* (%)	8 (44.4%)	17 (77.3%)	0.033
Depression, *N* (%)	5 (27.8%)	8 (36.4%)	0.564
**Clinical and laboratory findings at admission**			
Systolic BP, mmHg	154.78 ± 35.05	153.05 ± 25.78	0.817
Diastolic BP, mmHg	81.78 ± 17.68	88.05 ± 23.44	0.236
Heart rate, bpm	80.78 ± 15.32	73.82 ± 11.4	0.153
Oxygen saturation, %	97.66 ± 1.41	97.14 ± 2.04	0.555
Temperature, °C	36.42 ± 0.19	36.45 ± 0.22	0.989
Glycemia, mg/dL	125.17 ± 55.32	145.95 ± 52.36	0.128
Total cholesterol, mg/dL	200.28 ± 43.81	206.23 ± 49.45	0.734
Triglycerides, mg/dL	159.94 ± 88.51	165.86 ± 84.25	0.568
**Prior therapy**			
Antihypertensive drugs, *N* (%)	11 (61.1%)	13 (59.1%)	0.897
Antiplatelet drugs, *N* (%)	6 (33.3%)	8 (36.4%)	0.842
Anticoagulant drugs, *N* (%)	2 (11.1%) (VKA)	2 (9%) (4.5% VKA, 4.5% NOAC)	0.499
Statins, *N* (%)	4 (22.2%)	5 (22.7%)	0.970
**Clinical syndrome**			
Lacunar, *N* (%)	1 (5.6%)	5 (22.7%)	
Partial anterior circulation, *N* (%)	15 (83.3%)	15 (68.2%)	
Total anterior circulation, *N* (%)	–	–	
Posterior circulation, *N* (%)	3 (16.7%)	4 (18.1%)	
Multi-territorial, *N* (%)	–	3 (13.6%)	
**CT findings**			
Infarct volume, cm^3^	10.6 ± 15.17	23.19 ± 49.02	0.645

**Figure 2:**
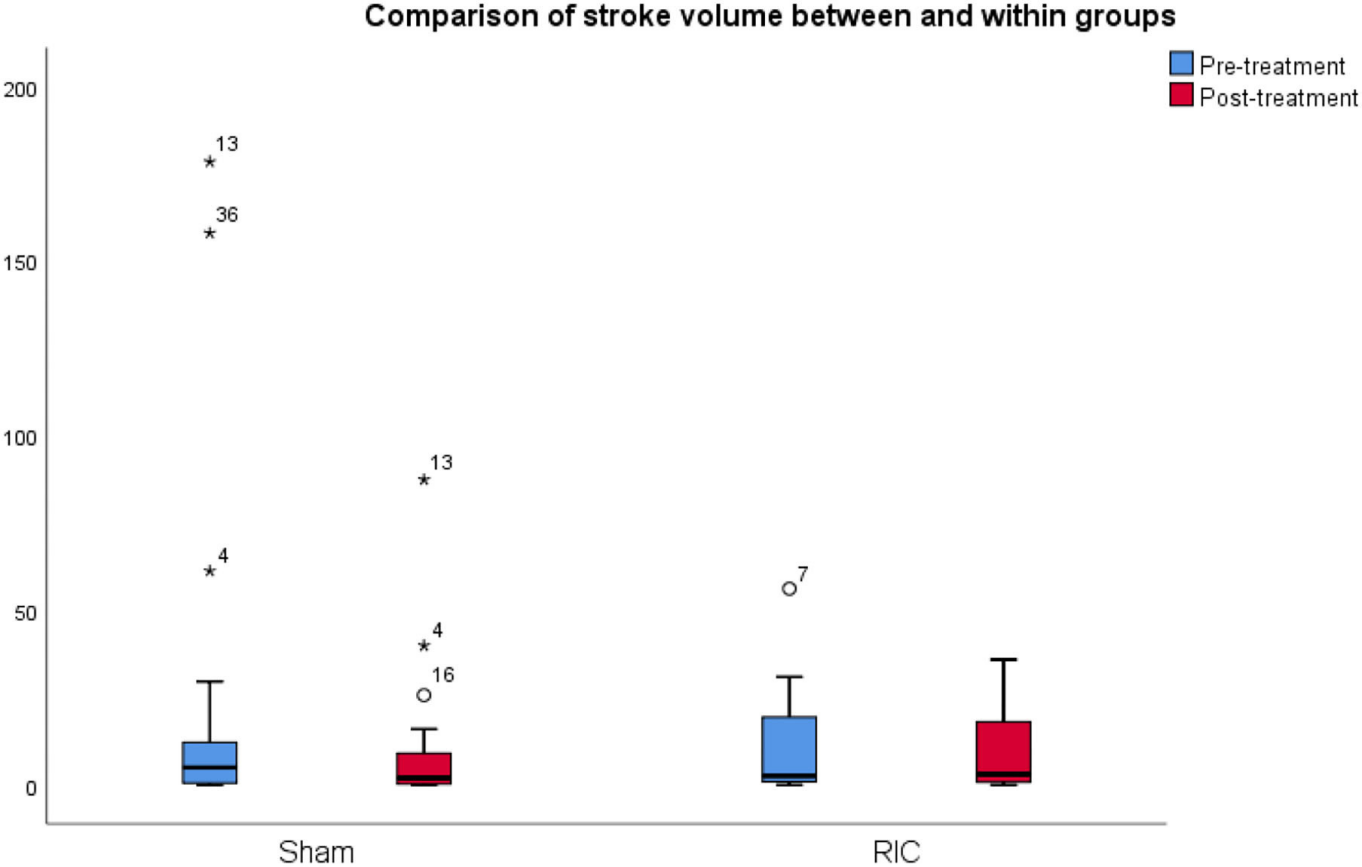
Comparison of infarct volume between and within groups. The mean infarct volume on CT at baseline and 6 months after stroke in the sham group and the RIC group. The median difference of the final infarct volume at 6 months compared to baseline was slightly larger in the sham group, but these results did not reach statistical significance (*p* = 0.4). One of the reasons for a better outcome in terms of stroke volume in the sham group might be the larger infarct volumes at admission as compared to the experimental group.

**Figure 3:**
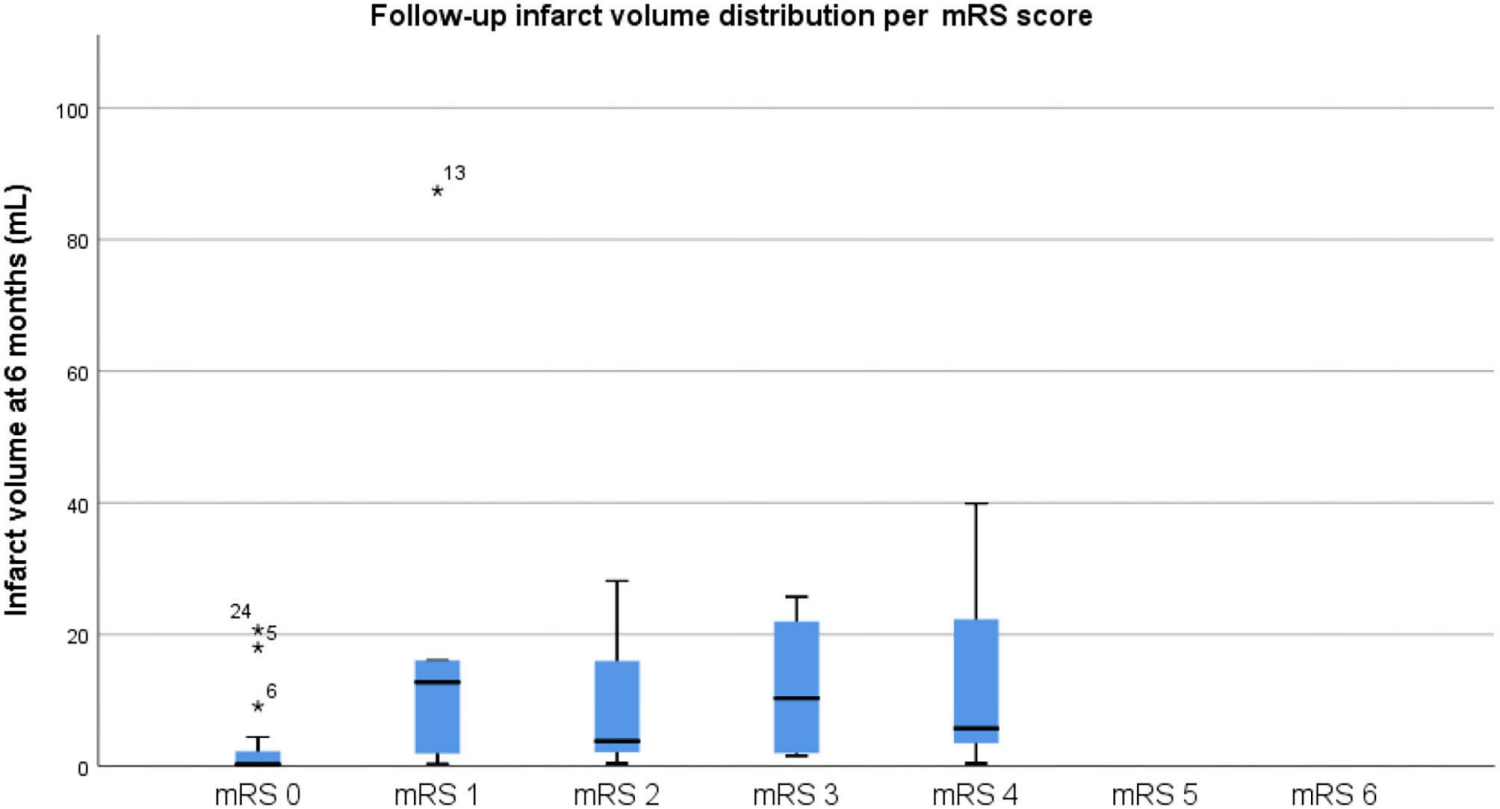
Final infarct volume distribution per mRS score. Please note that a larger final infarct volume was associated with increased mRS (*p* = 0.001), as expected.

Regarding the primary outcome, the mean infarct volume on CT at baseline was 23.19 cm^3^ in the sham group and 10.6 cm^3^ in the RIC group, whereas the final infarct volume was 10.35 cm^3^ in the sham group and 9.38 cm^3^ in the RIC group. The median difference of the final infarct volume at 6 months compared to baseline was slightly larger in the sham group, but these results did not reach statistical significance (*p* = 0.4). One of the reasons for a better outcome in terms of stroke volume in the sham group might be the larger infarct volumes at admission compared to the experimental group (Figure 2, Table 3). Another possible confounder is the baseline CT performed at days 3–4 (after RIC initiation), meaning that RIC could have already affected infarct volume, hence impacting the measure of infarct growth. As expected, larger final infarct volume was associated with increased mRS (*p* = 0.001) (Figure 3).

**Table 3:** Summary of primary and secondary outcomes in the two groups.

**Scale (score difference between 0 and 6 months)**	**Remote ischemic conditioning**						***p*-value (Mann-Whitney *U* test)**
	**SHAM**			**RIC**			
	**Minimum**	**Maximum**	**Median**	**Minimum**	**Maximum**	**Median**	
Infarct volume	−5.1	156	0.37	−5.4	20.3	0.29	0.4
NIHSS	0	11	5	1	8	5	0.1
mRS	0	4	1	0	4	1	0.3
Barthel	−80	0	−10	−60	100	−17.5	0.9
ADL	−8	0	−2	−6	10	−2.5	0.8
IADL	−8	2	−1	−6	8	−0.5	0.4
MoCA	−19	4	−2	−10	2	−3	0.2
PHQ-9	−12	17	0	−17	7	0	0.5

The details related to the secondary outcomes are listed in Table 3. The patients in the interventional group had slightly better recovery in terms of disability, as demonstrated by the differences in disability scores between admission and 6 months (e.g., the median difference score for Barthel was −10 in the sham group and −17.5 in the interventional group, for ADL −2 in the sham group and −2.5 in the interventional one), as well as cognitive performance (the median difference score for MoCA was −2 in the sham group and −3 in the interventional group), but none of these differences reached statistical significance. Nevertheless, the trend was similar in the two groups in terms of clinical stroke severity (median difference score for NIHSS = 5 for both groups) and depression (median difference score for PHQ-9 was 0 for both groups). Cognitive and depression scales could not be performed in 3 patients who were aphasic.

The mortality rate was 4.5% (one patient) in the sham group and 11.1% (two patients) in the RIC group. Recurrent ischemic stroke was 5.6% with one recurrent stroke in the RIC group, which occurred on day 14. There was no intracerebral hemorrhage.

Remote ischemic conditioning was well-tolerated and did not correlate with local (pain or bruising at the cuff side), cerebral (cerebral oedema, recurrence of stroke), or systemic (bedsores, urinary tract infection, bronchopneumonia, or death rate) complications.

## Discussion

To our knowledge, this is the first study employing RIC in AIS patients ineligible for reperfusion treatment, which evaluated the final infarct volume on CT scan and the functional outcome scales. We chose to evaluate the final infarct volume on CT scan because it is inexpensive and widely accessible, regardless of the economic status.

Our study indicates that RIC is safe, posing little risk for patients (i.e., no local or systemic events). It is also feasible since all patients finished the cycles of transient limb ischemia and the procedure was well-tolerated and inexpensive. We did not find any serious side effects.

Although RIC has been preliminarily investigated, the optimal protocol has not been found. In previous studies, there were different types of RIC procedures: different sites of limb conditioning (arm, leg, both arms, both legs); different number of cycles and duration of inflation/deflation; different timing of RIC; different frequency of inflation/deflation cycles (once or repeated different times per day) (13, 22, 23). We chose to cover the hyper acute-acute phase of AIS (inclusion within the first 24 h from the onset of symptoms/signs), by applying 5 cycles of inflation lasting 3 min, each followed by 5 min of reperfusion, twice daily, during the first 5 five days of hospitalization (in the morning and afternoon).

The neuroprotective effect of RIC supposedly acts in different ways, depending on the time of administration: when applied during the rapid therapeutic time window, RIC might minimize the side effects of sudden reperfusion (i.e., overproduction of reactive oxygen and nitrogen species); during the intermediate therapeutic time window, RIC is likely to enhance survival signaling pathways; during the delayed therapeutic window lasting for the first few days post stroke, RIC might promote synaptogenesis, angiogenesis, and neurogenesis (24). In our trial, RIC was started as soon as the patient arrived at the hospital and lasted for the first 5 days, during the intermediate and delayed therapeutic time window, acting during the post stroke reperfusion.

The primary outcome measure in our study was radiological, showing the difference in brain infarct volume between baseline and 6 months in both the experimental and control group. In the STAIR Group 2016 (Stroke Treatment Academic Industry Roundtable), the final infarct volume on brain imaging has been designated as a biomarker of early efficacy of a new treatment, being linked to the clinical outcome (25, 26). Furthermore, this outcome has been confirmed as a strong independent predictor of functional outcome in patients with ischemic stroke (27). In our study, the difference in infarct volume between baseline and 6 months was slightly larger in the sham group; however, this must be interpreted with caution considering the small number of patients enrolled and the larger infarct volume at baseline (days 3–4) in this group. Moreover, the smaller infarct size in the RIC group at baseline (days 3–4, after initiating RIC) might have been already influenced by RIC.

The clinical outcome measure was investigated as a second target using multiple validated scales: NIHSS, mRS, Barthel Index, IADL, ADL. ADLs refer to the most basic functions of living (eating, bathing, dressing, moving). All these reflect the quality of life of a stroke survivor. Impaired scores are associated with poorer medical health and increasing medical costs. Our results showed improvement in the disability scores at 6 months in the RIC group as compared to the sham group, but none of these findings reached statistical significance. Although the trends appear in favor of RIC, a larger efficacy trial is needed to certify this. In this regard, we draw attention to the larger ongoing phase III trials such as RESIST (NCT03481777), RECAST-3 (ISRCTN63231313), and REMOTE-CAT (NCT03375762) (28–30).

Montreal Cognitive Assessment is one of the most commonly used tests for cognitive status, with high sensitivity for detection of post stroke cognitive impairment. Our study found that MoCA was slightly higher in the RIC group, suggesting that RIC might improve cognitive function after stroke; however, this finding was not statistically significant. These results are consistent with those of a recent study conducted by Feng et al., which included 104 patients and demonstrated that RIC promotes improvement in cognition post stroke (31).

Post stroke depression is a common complication of stroke survivors. It is generally underdiagnosed and undertreated. Nevertheless, in our study, the depression PHQ-9 test was similar in the two groups (median difference score for PHQ-9 was 0 for both groups).

Our study has both strengths and limitations. The design of the study (i.e., double-blinded, randomized controlled trial) is a valuable asset. All patients had an AIS confirmed on CT scan, with follow-up infarct volume at 6 months as the primary outcome. By contrast, previous studies assessed the brain infarct volume on MRI (6–9). All our patients underwent the whole protocol of RIC, receiving all the cycles of cuff inflation. According to STAIR and STIR recommendations (18, 19), we evaluated the effects of RIC on the long clinical term (NIHSS, mRS) and the final infarct volume at 6 months. One of the most important limitations of the study resides in its unicentric enrollment, with a small sample size that possibly undermines the results. When baseline CT was performed, RIC might have already influenced the infarct size, possibly modifying the measure of infarct growth. We applied RIC manually although an automatic device would be more suitable for better completion of conditioning cycles and to document the treatment compliance. Moreover, we did not record the level of activity and rehabilitation after discharge and until follow-up, which might influence the functionality at 180-days and act as a confounding factor for the outcome. Another possible confounder is the sedentarism which was more frequent in the sham group at baseline.

## Conclusion and Future Directions

Remote ischemic conditioning is a promising therapy, inexpensive, well-tolerated, supposedly neuroprotective. It might show good results in terms of functionality, and it could be easily implemented as a routine procedure in pre-hospital transport, emergency room, hospitals, or even intensive care units. Further randomized controlled trials with a larger sample size and optimized RIC protocols are required in order to establish and certify the effects of RIC in AIS. Another focal point should be the location of RIC delivering and/or brain lesion, which might influence the outcome of RIC in AIS.

## Data Availability Statement

The original contributions presented in the study are included in the article/supplementary material, further inquiries can be directed to the corresponding author/s.

## Ethics Statement

The studies involving human participants were reviewed and approved by the Ethical Committee of Emergency Clinical Hospital Bucharest. The patients/participants provided their written informed consent to participate in this study.

## Author Contributions

BP is the coordinating investigator and scientific supervisor of the study. AP realized the study design, data collection, and manuscript writing. ET analyzed the CT scans. DT performed the statistical analysis and manuscript writing. DS did the editing assistance. All authors contributed to the article and approved the submitted version.

## Conflict of Interest

The authors declare that the research was conducted in the absence of any commercial or financial relationships that could be construed as a potential conflict of interest.

## Publisher's Note

All claims expressed in this article are solely those of the authors and do not necessarily represent those of their affiliated organizations, or those of the publisher, the editors and the reviewers. Any product that may be evaluated in this article, or claim that may be made by its manufacturer, is not guaranteed or endorsed by the publisher.
